# Balance performance of healthy young individuals in real versus matched virtual environments: a systematic scoping review

**DOI:** 10.3389/fnhum.2024.1422581

**Published:** 2024-07-29

**Authors:** Simon Schedler, Klaus Gramann, Mathew W. Hill, Thomas Muehlbauer

**Affiliations:** ^1^Division of Movement and Training Sciences/Biomechanics of Sport, University of Duisburg-Essen, Essen, Germany; ^2^Department of Biological Psychology and Neuroergonomics, TU Berlin, Berlin, Germany; ^3^School of Software, University of Technology Sydney, Sydney, NSW, Australia; ^4^Centre for Physical Activity, Sport and Exercise Sciences, Coventry University, Coventry, United Kingdom

**Keywords:** postural control, balance performance, virtual reality, head-mounted display, healthy youth, healthy young adults

## Abstract

**Background:**

Due to technological advancements and the development of consumer-oriented head mounted displays (HMDs), virtual reality (VR) is used in studies on balance performance and balance trainability more and more frequently. Yet, it may be assumed that balance performance is affected by the physical characteristics of the HMD (e.g., weight) as well as by the virtual visual environment. Moreover, it has been shown that balance is age-dependent with children and adolescents showing worse performances compared to young adults, which may also affect their balance performance in virtual environments.

**Objectives:**

The present systematic scoping review aims to provide an overview on the current evidence regarding balance performance of healthy, young individuals (6–30 years) in real and matched virtual environments.

**Methods:**

A systematic literature search in the electronic databases PubMed, Web of Science, and SPORTDiscus (from their inception date to February 2024) resulted in 9,554 studies potentially eligible for inclusion. Eligibility criteria were: (i) investigation of healthy, young individuals (6–30 years), (ii) balance assessment in the real and a matched virtual environment, (iii) use of a fully immersive HMD, (iv) reporting of at least one balance parameter. A total of 10 studies met the predefined inclusion criteria and were thus included in this review. All studies were conducted with healthy, young adults (19–30 years).

**Results:**

Five studies assessed static balance, four studies quantified dynamic balance, and one study measured static as well as dynamic balance performance. In healthy young adults, static balance performance was similar with and without VR during simple standing tasks (e.g., two-legged stance), but worse in VR during more challenging tasks (e.g., one-legged stance). Concerning dynamic balance, four out of five studies reported worse performance in VR, while one study did not find differences between visual environments. Most importantly, none of the studies investigating healthy children (6–12 years) and/or adolescents (13–18 years) met the predefined inclusion criteria.

**Conclusion:**

In healthy young adults, balance performance seems to be affected by VR only during challenging static (e.g., one-legged stance) as well as during dynamic balance tasks. The underlying causes remain unclear, but factors such as perceived presence in VR, a shift in sensory organization and/or perceptual distortion may play a role. Of particular importance is the finding that there is a void in the literature on the influence of VR on balance performance of healthy children and adolescents.

## Introduction

In recent years, the use of immersive virtual reality (VR) has become increasingly popular in sciences due to advancements in the underlying technology, reduced acquisition costs, and a constantly growing spectrum of applications. A literature search in the online database PubMed using the term “virtual reality” revealed that over the last two decades the number of available peer-reviewed publications relating to this topic has increased from 1,188 in 2003 to 21,978 in 2023. An important step in the technological progress of VR-systems was the development of modern high-resolution head mounted displays (HMDs) over the last decade. Previously used equipment often included (multiple) large, stationary screens to apply a visual stimulus to a subject, thus limiting the possibility to synchronize visual input with the subject’s head movements and leaving them unable to move freely in the virtual environment. For instance, in a study by Hollman et al. (2006) participants had to walk on a treadmill while looking at a moving, continuous virtual corridor projected onto a curved screen placed in front of the treadmill. On the one hand, such an approach limits immersion (i.e., the devices’ technological capability to deliver lifelike experiences) as for example physical reality was not completely shut out (Slater and Wilbur, 1997). On the other hand, participants’ perceived presence (i.e., the sense of really being in the virtual environment) may be restricted as they could not move freely in VR (Slater and Wilbur, 1997). In contrast, modern HMDs are relatively lightweight devices which provide subjects with an almost lifelike field of view and visual as well as auditory stimuli which are constantly aligned with the individual’s (head) movements (Garner, 2018) and it has indeed been shown that they provide greater immersion (Rose et al., 2018) and higher presence (Shu et al., 2019) than less sophisticated VR-tools.

Exposing individuals to VR using modern HMDs thereby enables researchers to investigate an individual’s behavior under almost real life (visual and auditory) conditions, such as walking across a crowded pedestrian crossing or standing in an open-air elevator while physically remaining in the laboratory and applying appropriate test equipment (e.g., motion capturing, force plate; Koilias et al., 2020; Bzduskova et al., 2022). Thus, VR may help to overcome the everlasting challenge of weighing up the pros and cons when deciding whether to conduct a lab- or a field-based test. Consequently, it is not surprising that VR is also used in postural control research more and more frequently as indicated by an almost threefold increase of yearly publications indexed in PubMed from 2013 (*N* = 34 studies) to 2023 (*N* = 92 studies) when searching for “virtual reality postural control.”

In this regard, studies using HMD-driven VR have focused on balance assessments (Rosiak et al., 2024) as well as on balance training (Lubetzky et al., 2022). During several balance assessments [e.g., Sensory Organization Test (Nashner and Peters, 1990)] for example, researchers apply test conditions where visual input is manipulated (e.g., by a moving visual stimulus) to assess the contribution of different afferents (i.e., visual, vestibular, somatosensory) for the control of balance. However, these tests usually require sophisticated equipment which is costly and stationary. To address this problem, researchers have successfully adapted the test conditions using HMDs (Wittstein et al., 2020; Moon et al., 2021). Additionally, HMDs allow researchers to use an almost infinite number of configurations regarding the design of a visual perturbation (e.g., amplitude, direction, frequency) and/or the visual environment and thereby may help in identifying individuals with balance deficits and/or at risk of falls (Soltani and Andrade, 2020).

Concerning balance, one group of scientific interest are children and adolescents. More specifically, it has been shown that, compared to adults, they show poorer balance performance, even if they are generally healthy (Balogun et al., 1994; Granacher et al., 2011; Schedler et al., 2021). The poorer balance performance can most likely be attributed to the immaturity of their postural control system (e.g., vestibular function, cerebellar function), which does not develop until early adulthood (Gouleme et al., 2014, 2018). Similarly to older individuals, lower balance performance in youth is accompanied with an increased risk of falls (Tang et al., 2021) and/or sustaining sports-related injuries (Emery, 2003). Consequently, balance assessments as well as balance training are particularly important in the healthy youth. Besides the aforementioned potential benefits of VR for postural control research, using virtual environments may have additional advantages such as higher motivation and entertainment for the younger populations. Moreover, children are known to rely more on vision than young or older adults as their vestibular system is still developing (Hirabayashi and Iwasaki, 1995; Steindl et al., 2006). Therefore, one could assume that the effects of the virtual environment on balance performance could vary depending on age.

Despite the aforementioned potential benefits of VR for postural control research, it has to be considered that balance performance might be affected when applying VR using HMDs. More precisely, one could argue that balance performance when viewing a virtual environment may not reflect an individual’s capability to balance in the real environment. In fact, some studies have addressed this question resulting in equivocal findings. For young adults, Asslander and Streuber (2020) showed a comparable body sway during two legged-stance on a fixed as well as on a moving platform while viewing the real or a virtual replica of the laboratory. In contrast, Peterson et al. (2018) observed significantly worse performances (i.e., more failures, lesser beam passes, slower gait speed) when young adults balanced on a virtual compared to a real balance beam. Further and with respect to the aforementioned age-related differences in balance performance in youth, it may be supposed that VR may have different effects on healthy children’s, adolescents’, and/or young adults’ balance performance. Such age-related differences of the influence of VR on balance would however be of major importance for researchers as well as for practitioners (e.g., physical therapists, coaches) to be able to design appropriate test and training conditions for different age groups when VR is used to assess and/or train balance. For instance, if VR has age-dependent effects on balance performance in healthy children, adolescents and young adults, test conditions (i.e., during balance assessment) and balance exercises (i.e., during balance training) may have to be adapted when VR is used.

Therefore, the aim of the present systematic scoping review was to aggregate findings on balance performance in healthy children, adolescents, and young adults when assessed in real and matched virtual environments.

## Methods

The present systematic scoping review was conducted according to the PRISMA Extension for Scoping Reviews (PRISMA-ScR; Tricco et al., 2018). The procedure followed a preassigned protocol, which was however neither registered nor published. The protocol can be provided by the corresponding author if requested.

### Search strategy

To identify relevant research articles, a systematic literature search in the electronic databases PubMed, Web of Science, and SPORTDiscus was performed using the following Boolean search term: (“virtual reality” OR “VR” OR “virtual” OR “augmented reality” OR “AR” OR” virtual environment” OR “head mounted display” OR” HMD” OR “immersive”) AND (“balance” OR “postural control” OR” postural stability” OR” posturography” OR “equilibrium” OR “posture” OR “beam”) NOT (“patients” OR “rehabilitation” OR “syndrome” OR “cerebral palsy” OR “stroke” OR “disorder” OR “injury” OR “disease” OR “elderly” OR “seniors”). The search was limited to English full texts investigating the human species published from the inception date of the respective database to March 2024. After duplicates were removed, two authors (SS, TM) independently screened the titles of potentially eligible studies sequentially according to the predefined inclusion and exclusion criteria. Subsequently, the abstracts and full-texts of the remaining articles were read and assessed for eligibility by both authors. Disagreements were solved through discussion and consensus. No screening software was used during the whole inquiry.

### Study selection criteria

Study selection was conducted according to several predefined inclusion and exclusion criteria (Table 1). Eligible for inclusion were English full-texts investigating healthy young individuals (6–30 years), which assessed balance performance in the real and a matched virtual environment provided through a fully immersive HMD. Additionally, only cross-sectional studies and intervention studies providing baseline data were considered for inclusion. With respect to balance performance, studies were selected if they reported at least one parameter of either static or dynamic balance based on the definitions provided by Shumway-Cook and Woollacott (2007). Concerning static balance, the preferred outcome was postural sway velocity, while regarding dynamic balance it was gait velocity. An overview on the preferred and alternative outcomes is provided in Table 2.

**Table 1:** Inclusion and exclusion criteria applied during the literature search.

	Inclusion criteria	Exclusion criteria
Document	English full-text	Abstracts, Conference Papers, non-English full-text
Study group	Healthy individuals	Athletes, patients (e.g., morbus parkinson, cerebral palsy)
Age	6–30 years	>30 years
Study design	Cross-sectional, Intervention	Case report, Observational, Intervention without reporting baseline data, Systematic Review
Hardware used for virtual, visual stimulus	Fully immersive, stereoscopic head-mounted-display (e.g., HTC Vive)	Screen-based devices (e.g., CAVE system), smartphone-based devices (e.g., Google cardboard)
Virtual visual environment	Resemblance of real visual environment (e.g., laboratory)	Fictional visual environment (e.g., city, forest, transformer station)
Outcome	At least one parameter of balance performance (e.g., sway velocity)	Brain activity, psychometric measures

**Table 2:** Overview of the preferred and alternative outcomes for static and dynamic balance.

	Preferred outcome	Alternative outcomes
Static balance performance	Postural sway velocity	Postural sway velocity in medio-lateral direction Postural sway velocity in anterio-posterior direction Postural sway path Root-mean-square error of postural sway
Dynamic balance performance	Gait velocity	Failures Number of steps

### Data extraction

Data of included studies was extracted and specified according to author(s), year of publication, number, age, and sex of participants, balance assessment and outcomes, VR hardware (i.e., HMD used), and results. Relating to participants’ age, studies were categorized as investigating children (<12 years), adolescents (12–18 years), and/or young adults (18–30 years). Further, depending on the respective balance assessment(s), studies were categorized as investigating static (i.e., maintain balance while body is stationary) and/or dynamic (i.e., maintain balance while body is moving) balance performance.

## Results

### Study selection

**Figure 1:**
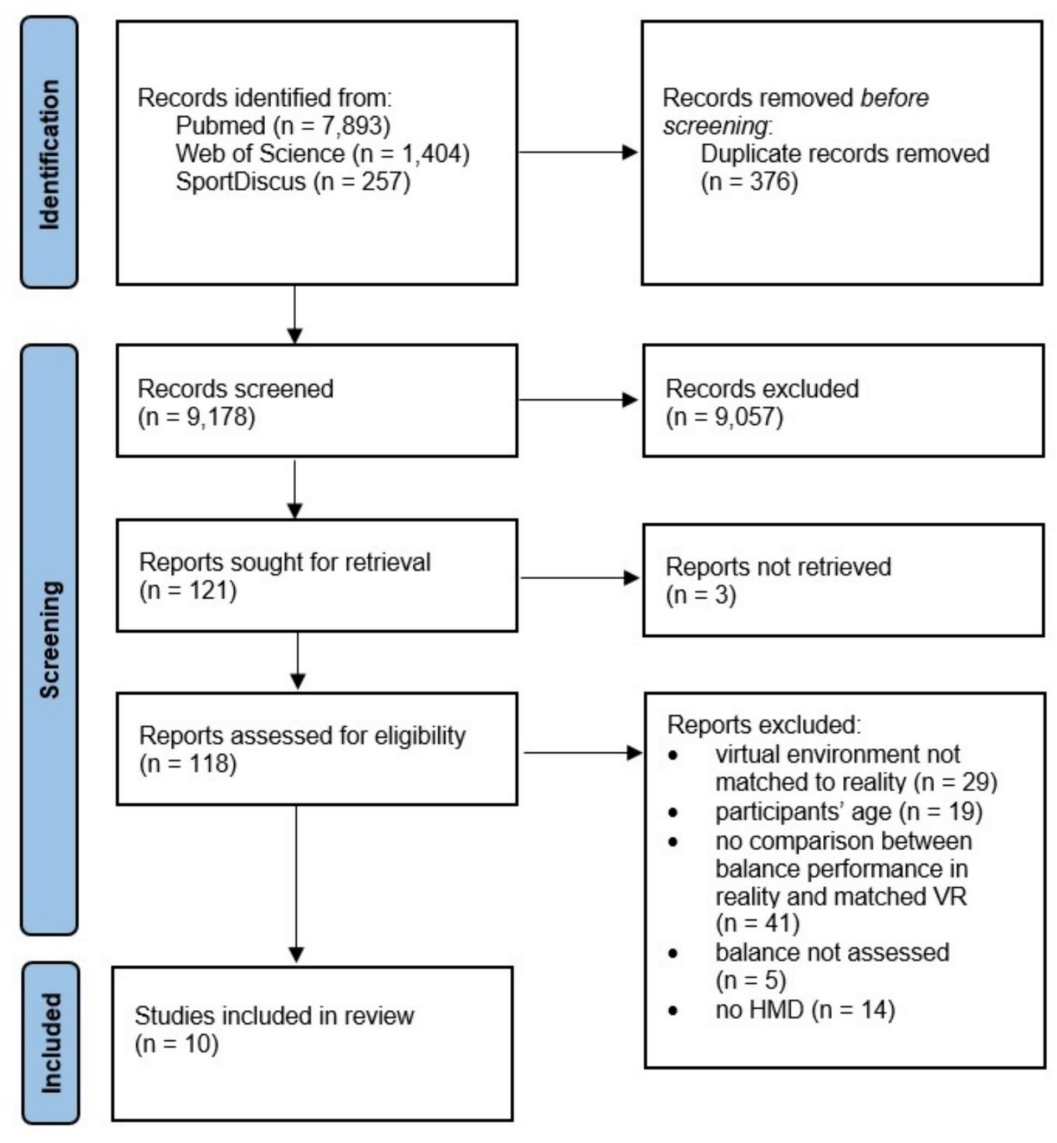
PRISMA flow chart showing the different stages of the literature search.

A complete overview of the selection process following the literature search is provided in the Flow chart depicted in Figure 1. Initially, the systematic literature search revealed 9,554 studies potentially eligible for inclusion in this review. After removing duplicates (*n* = 376), 9,057 studies were excluded based on their title. Of the remaining 121 records, 118 were retrieved and assessed for eligibility. The majority of these studies was excluded, for the following reasons: (i) the virtual environment was not matched to the real environment (*n* = 29), (ii) the age of included participants (*n* = 19), (iii) balance performance was not compared between real and matched virtual conditions (*n* = 41), (iv) no balance assessment (*n* = 5), (v) no HMD was used (*n* = 14). Consequently, the systematic literature search resulted in a total of 10 studies (Kelly et al., 2008, 2019; Cleworth et al., 2012; Ida et al., 2017; Peterson et al., 2018; Asslander and Streuber, 2020; Pastel et al., 2020; Liang et al., 2021; Ketterer et al., 2022) which were included in this systematic scoping review.

### Study characteristics

The main characteristics of the included studies are presented in Table 3. None of the included studies investigated balance performance in a real and a matched virtual environment in healthy youth; neither in children nor in adolescents. Thus, all of the included studies were conducted with healthy young adults. Altogether, 193 (93 females, 100 males) healthy young adults (18–30 years) were investigated. Five studies (Kelly et al., 2008, 2019; Cleworth et al., 2012; Liang et al., 2021; Ketterer et al., 2022) assessed static balance performance, four studies (Ida et al., 2017; Peterson et al., 2018; Boroomand-Tehrani et al., 2020; Pastel et al., 2020) measured dynamic balance performance, and one study (Asslander and Streuber, 2020) recorded static as well as dynamic balance performance. Eight different HMDs were used in the included studies with the HTC Vive Pro (*n* = 2; Asslander and Streuber, 2020; Liang et al., 2021) and the HTC Vive Pro Eye (*n* = 2; Pastel et al., 2020; Ketterer et al., 2022) being the ones used most frequently. The other employed HMDs were Virtual Research V8 (*n* = 1; Kelly et al., 2008), Vuzix iWear (*n* = 1; Ida et al., 2017), Sensics piSight (*n* = 1; Cleworth et al., 2012), Oculus Rift DK1 (*n* = 1; Boroomand-Tehrani et al., 2020), Oculus Rift DK2 (*n* = 1; Peterson et al., 2018), and HTC Vive (*n* = 1; Kelly et al., 2019). All of these HMDs are PC-powered and thus cable-bound. In accordance with the predefined inclusion criteria, participants viewed a virtual resemblance of the real surrounding (e.g., laboratory) during balance assessment under VR conditions.

**Table 3:** Characteristics of the included studies examining balance performance of healthy, young individuals in real and matched virtual environments.

Reference	Study group (age, sex)	Balance test(s) (type of balance)	HMD	VR-scenario	Balance outcomes	Results
Kelly et al. (2008)	8 young adults (N/A; 2 *f*, 6 *m*)	Side-by-side, heel-to-toe, and one-legged stance (static)	Virtual Research V8	Virtual Laboratory	Body sway (analyzed via head movements)	Balance performance in VR significantly worse compared to REAL, but only in more demanding tasks (i.e., heel-to-toe, one-legged stance)
Cleworth et al. (2012)	18 young adults (19–28 years old; 11 *f*, 7 *m*)	Two-legged stance on a force platform at 0.8 m and 3.2 m (static)	Sensics piSight	Virtual Laboratory	RMS of CoP in A/P and M/L directions MPF of CoP in A/P and M/L directions Mean position of CoP in A/P direction	No significant differences between balance performances in VR and REAL
Ida et al. (2017)	10 young adults (27.3 ± 4.7 years; 5 *f*, 5 *m*)	Leg lifting to avoid an approaching obstacle while standing on a force platform (dynamic)	Vuzix iWear VR920	Virtual Laboratory	CoP sway path in M/L direction sEMG Accelerometry	Balance performance in VR significantly worse compared to REAL
Peterson et al. (2018)	19 young adults (23 ± 4 years; 9 *f*, 10 *m*)	Heel-to-toe beam walking on a balance beam (305 × 3.8 × 2.5 cm) (dynamic)	Oculus Rift Development Kit 2	Virtual Laboratory (participants also walked over the physical beam in the VR conditions)	Failures (i.e., stepping off the beam) Beam passes Gait speed	Balance performance in VR significantly worse compared to REAL
Kelly et al. (2019)	32 young adults (19.78 ± 1.93 years; 16 *f*, 16 *m*)	One-legged stance facing a door from close (0.85 m) and far (3 m) distance (static)	HTC Vive	Virtual laboratory	RMS of body sway Sway velocity Romberg quotients (all analyzed via head movements)	Balance performance in VR significantly worse compared to REAL
Asslander and Streuber (2020)	10 young adults (25.4 ± 4.7 years; 5 *f*, 5 *m*)	Two-legged stance on custom built platform (static) Two-legged stance on custom built moving platform (dynamic)	HTC Vive Pro	Virtual Laboratory	Sway velocity	No significant differences between balance performances in VR and REAL
Boroomand-Tehrani et al. (2020)	22 young adults (22.8 ± 1.8 years; 12 f, 12 m)	Beam walking on a balance beam (300 × 3.8 × 10 cm) (dynamic)	Oculus Rift Development Kit 1	Virtual Laboratory	Step success Steps before first fail Time to first fail	Balance performance in VR significantly worse compared to REAL
Pastel et al. (2020)	20 young adults (21.6 ± 1.6 years; 7 *f*, 13 *m*)	Beam walking on a balance beam (400 × 10 × 4 cm) (dynamic)	HTC Vive Pro Eye	Virtual Laboratory with different grades of body representation (i.e., whole body, whole body except feet, whole body except feet and legs, no body); (participants also walked over the physical beam in the VR conditions)	Time to completion Failures (i.e., stepping off the beam) No. of steps	Balance performance in VR significantly worse compared to REAL
Liang et al. (2021)	34 young adults (26.5 ± 6.3 years; 23 *f*, 11 *m*)	Two-legged (i.e., standard, narrow, tandem) and one-legged stance wearing a tracker (static)	HTC Vive Pro	Virtual Laboratory	CoP sway path in A/P and M/L directions CoP velocity in A/P and M/L directions	Balance performance in VR significantly worse compared to REAL, but only in more demanding tasks (i.e., narrow tandem, one-legged stance)
Ketterer et al. (2022)	18 young adults (24.1 ± 2.0 years; 10 *f*, 8 *m*)	Two-legged stance (static)	HTC Vive Pro Eye	Virtual Laboratory	CoM sway assessed via 3D movement analysis	No significant differences between balance performances in VR and REAL

A/P, anterior–posterior, CoM, Centre of mass; CoP, Centre of pressure; EC, eyes closed; EO, eyes opened; f, female; HMD, Head-Mounted-Display; m, male; M/L, medial-lateral; MPF, mean power frequency; N/A, not applicable; No., Number; RMS, root mean square; sEMG, surface electromyography; VR, virtual reality.

Two of the included studies assessed virtual reality sickness using either the “Motion Sickness Assessment Questionnaire (MSAQ)” (Peterson et al., 2018), or the “Simulator Sickness Questionnaire (SSQ)” (Asslander and Streuber, 2020). Further, one study (Asslander and Streuber, 2020) used the “iGroup Presence Questionnaire (IPQ)” and one study (Boroomand-Tehrani et al., 2020) used the” Presence Questionnaire” to measure the degree of perceived presence in the virtual reality scenario.

### Static balance performance under real versus matched virtual conditions

Three studies (Cleworth et al., 2012; Asslander and Streuber, 2020; Ketterer et al., 2022) did not find significant differences between static balance performance under real when compared to matched virtual conditions. Another three studies (Kelly et al., 2008, 2019; Liang et al., 2021) observed worse performances in VR compared to the real condition, but only when balance task difficulty was increased (e.g., by reducing the base of support). No study detected worse static balance performance in the real compared to the virtual environment.

### Dynamic balance performance under real versus matched virtual conditions

Comparing participants’ dynamic balance performance under real versus matched virtual conditions, four studies (Ida et al., 2017; Peterson et al., 2018; Boroomand-Tehrani et al., 2020; Pastel et al., 2020) observed worse performances in the virtual compared to the real environment. In contrast, one study (Asslander and Streuber, 2020) did not find significant differences in dynamic balance performances between real and VR conditions. There was no study which found worse dynamic balance performance in the real compared to the virtual environment.

## Discussion

The results of the present systematic scoping review can be summarized as follows: (i) in the majority of the included studies (i.e., seven out of ten) balance performance was at least to some extent worse when assessed under virtual compared to real conditions, (ii) detrimental effects of virtual reality were evident during demanding static (e.g., one-legged stance) and dynamic balance tasks, (iii) no study investigating children and/or adolescents met the inclusion criteria.

### The influence of VR on static balance performance

At first glance, the results of the influence of VR on static balance performance in healthy young individuals seem to be inconsistent as three studies (Cleworth et al., 2012; Asslander and Streuber, 2020; Ketterer et al., 2022) did not find differences between balance performance in VR compared to the real environment, whereas three studies (Kelly et al., 2008, 2019; Liang et al., 2021) reported worse performance when individuals balanced while being exposed to VR. With respect to the latter studies, Kelly et al. (2008) assessed balance performance during three stance conditions (i.e., side-by-side, heel-to-toe, one-legged stance) while viewing a real room as well as a virtual replica in eight undergraduate students (six males, two females). Significantly increased sway in the virtual compared to the real visual environment was only observed during the heel-to-toe and the one-legged stance, but not during the easier side-by-side stance. Similarly, Liang et al. (2021) reported significantly larger body sway in VR compared to the real visual environment during tandem- and one-legged-stance, but not during a standardized two-legged-stance and side-by-side standing in 34 young adults (11 males, 23 females; mean age: 26.5 years). Lastly, in the study by Kelly et al. (2019), who also detected worse performances in 32 young adults (16 males, 16 females, mean age: 19.78 years) in the virtual compared to the real environment, participant’s balance was only assessed during the more difficult one-legged stance. In contrast, the authors of the studies which reported similar balance performances in the real and a matched virtual environment assessed balance during two-legged standing (Cleworth et al., 2012; Asslander and Streuber, 2020; Ketterer et al., 2022), which is relatively easy to perform, especially for healthy young individuals. Actually, these findings agree with those of Kelly et al. (2008) and by Liang et al. (2021) and overall the present analysis indicates that there is a task-specific effect of VR on static balance performance in healthy young adults in the age range between 19 and 30 years.

Based on these results, one could argue that the type of visual input (i.e., real vs. virtual) has little relevance during simple balance tasks (e.g., two-legged stance), which can also easily be performed when visual input is removed. Yet, during more challenging conditions (e.g., one-legged stance) studies have found that visual input becomes more important (Hazime et al., 2012; Tomomitsu et al., 2013). The results of the included studies however indicate that balance performance decreases in the virtual condition during challenging tasks even though visual input is available. Thus, despite the high fidelity of modern HMDs, visual input may still be perceived as being inappropriate or unreliable when viewing a virtual replica of the real environment. This could be related to altered depth perception in VR which, although it has significantly improved in modern HMDs, does still not match the distance estimates made in the real environment (Kelly, 2023). As a result, individuals may rely more strongly on somatosensory and/or vestibular input to keep their balance. Nonetheless, it has to be mentioned that participants in the included studies were still able to successfully perform the requested, more challenging balance tasks in the virtual environment.

Further, performance decreases were only observed in some of the included studies (Kelly et al., 2008, 2019; Liang et al., 2021) and even within some of these studies (Kelly et al., 2008; Liang et al., 2021) they were limited to certain test conditions. This indicates that balance performance is not affected by the physical characteristics of the HMD (e.g., mass), which corresponds to the findings of Mihalik et al. (2008) who could not find differences when participants performed different stance variations with eyes closed while wearing or not wearing a HMD.

### The influence of VR on dynamic balance performance

Four of the included studies (Peterson et al., 2018; Boroomand-Tehrani et al., 2020; Pastel et al., 2020; Ida et al., 2022) reported decreased dynamic balance performance when healthy, young adults viewed a virtual replica of the real surrounding indicating a detrimental effect of VR on dynamic balance performance. This is in contrast to the findings of Asslander and Streuber (2020) who could not find differences in body sway of 10 young adults (5 males, 5 females; mean age: 25.4 years) following pseudo-randomized perturbations while standing on a tilting-platform and viewing the real or a virtual replica of the laboratory. One possible explanation could relate to methodological differences between these studies as participants actively moved in the virtual environment in three studies (Peterson et al., 2018; Boroomand-Tehrani et al., 2020; Pastel et al., 2020) or at least reacted (i.e., leg-lifting) to a moving visual stimulus (i.e., approaching obstacle; Ida et al., 2017), while visual environment stayed relatively stable in the other study (Asslander and Streuber, 2020), in which participants responded to a perturbation of the support surface.

Peterson et al. (2018), Boroomand-Tehrani et al. (2020), and Pastel et al. (2020) compared beam walking performance on a physical balance beam while viewing the real environment or a virtual replica of it and observed worse performances (e.g., slower gait velocity) in the VR condition in 19 (10 males, 9 females; mean age: 23 years), 20 (13 males, 7 females; mean age: 23.6 years), and 24 young adults (12 males, 12 females; mean age: 22.8 years), respectively. Previous studies (Horsak et al., 2021, 2023) have already shown that healthy adults adopt a more cautious gait strategy (e.g., slower gait velocity, longer time in double support) when walking in a virtual environment, perhaps as a consequence of a sensory conflict induced by the virtual visual condition even when it resembles the real environment. This effect may have been amplified by the increased task difficulty (i.e., beam walking) in the included studies (Peterson et al., 2018; Boroomand-Tehrani et al., 2020; Pastel et al., 2020). In fact, participants in the study of Pastel et al. (2020) perceived beam walking in the virtual environment as being more difficult than in the real environment.

Concerning the effects of moving in VR, an interesting finding has been reported by Ketterer et al. (2022). These authors found no differences between static balance performance when viewing the real environment or a virtual replica in 18 young adults (8 males, 10 females; mean age: 24.1 years). However, after participants were allowed to move freely in VR for 3 min to accustom themselves with this visual condition, performance significantly decreased. This finding may either indicate that moving in VR indeed causes a shift in sensory organization and/or movement strategy or that factors such as the time spent in VR also affect postural control, for instance due to eye strain.

Further, body representation may be an important aspect to successfully perform a rather challenging balance task like beam-walking in VR. However, in two of the studies (Peterson et al., 2018; Pastel et al., 2020) participants were provided with an avatar in the virtual environment. Even more important, Pastel et al. (2020) compared individuals’ performances not only between the real and the virtual environment, but also between different levels of body representation in VR (i.e., whole body, whole body except of feet, whole body except of feet and legs, no body), that were synchronized to participants’ movements. Although the best balance performance in VR was achieved with whole body representation, it was still worse compared to the real condition. Thus, although the level of body representation seems to affect balance performance in VR, it cannot fully explain decreased performance in VR. However, it has to be mentioned that the avatars used were not entirely matched to participants regarding for instance stature, clothes, or shoes worn.

## Future directions

The present systematic scoping review revealed several issues that should be addressed in future studies. First and most importantly there is an urgent need for studies on the influence of HMD-driven VR on balance performance in healthy children and adolescents. Previous studies (Balogun et al., 1994; Gouleme et al., 2014, 2018) on the development of postural control have shown age-related differences between children’s, adolescents’ and young adults’ balance performances. Children, for example rely more heavily on visual input to control their posture and thus maintain their balance (Hirabayashi and Iwasaki, 1995; Steindl et al., 2006). Therefore, the effects of VR on balance performance may be stronger in children than young adults.

Two parameters potentially affecting balance performance in VR are an individuals’ perceived presence when immersed in the virtual environment (Asslander and Streuber, 2020) as well as virtual reality sickness (e.g., dizziness, nausea; Chang et al., 2020). However, presence was only assessed in two (Asslander and Streuber, 2020; Boroomand-Tehrani et al., 2020) of the included studies. Similarly, only two studies (Peterson et al., 2018; Asslander and Streuber, 2020) recorded proxies of virtual reality sickness. Future studies should analyze how these psycho-physical variables are associated with balance performance in healthy young individuals when viewing a virtual environment through a HMD.

The findings presented in this review are limited to immediate effects of VR matched to the real environment on balance performance. However, there is evidence that the time spent in VR affects balance performance in VR (Ketterer et al., 2022) as well as in the real environment once the HMD is taken off (Akizuki et al., 2005). This relationship should be investigated in future studies as it is particularly important with respect to VR-assisted balance training. Additionally, it might also be interesting to investigate long-term (training/de-training) effects of VR on balance performance as well as the effects different virtual environments. In this regard, one could suppose that balance may be affected more strongly when the virtual environment does not correspond to the real environment.

All of the HMDs used in the included studies were cable-bound and connected to a PC. This may have affected balance performance as well as perceived presence as the cords would probably move, especially in dynamic conditions, which participants presumably sensed. The latest generation of consumer-oriented HMDs (e.g., Meta Quest 3, HTC VIVE Focus 3) are standalone devices, which should provide users with an even more realistic experience when immersed in virtual reality. Consequently, future studies should investigate their effects on balance performance in healthy young populations.

Lastly, previous research (Esteves et al., 2022; Deodato et al., 2023) has also shown that unilateral injuries may not only affect balance performance of the injured but also of the actually healthy limb, perhaps due to a general reorganization of balance control. Future studies could investigate whether VR provides benefits for research within this topic, as it could for instance be used as a training tool.

## Limitations

There are a few limitations which have to be addressed. First, reporting biases cannot be assessed as the present protocol has not been registered or published in advance. Second, the presented findings only apply to healthy young individuals, who balanced under real and matched virtual conditions using modern fully immersive HMDs. Consequently, it remains unclear how different virtual environments affect balance performance.

## Conclusion

The present systematic scoping review revealed that healthy, young adults’ balance performance is affected by VR using modern, fully immersive HMDs during demanding static as well as during dynamic balance tasks, even when the virtual scene resembles the respective real visual environment. Currently, the underlying mechanisms remain unclear and should therefore be investigated in future studies. Factors such as perceived presence in VR as well as a shift in sensory organization due to the virtual visual environment could potentially play a role. More importantly, concerning balance there is a serious lack of studies on the use and effects of HMD-driven VR in healthy children and adolescents, which are a group of special interest due to their maturing postural control system.

## Data availability statement

The original contributions presented in the study are included in the article/supplementary material, further inquiries can be directed to the corresponding author.

## Author contributions

SS: Conceptualization, Data curation, Formal analysis, Investigation, Methodology, Visualization, Writing – original draft, Writing – review & editing. KG: Methodology, Writing – original draft, Writing – review & editing. MH: Methodology, Writing – original draft, Writing – review & editing. TM: Conceptualization, Methodology, Supervision, Writing – original draft, Writing – review & editing.
